# Refractory Neonatal Apnea Revealing Congenital Central Hypoventilation Syndrome: Improved Outcome through Early Multidisciplinary Intervention

**DOI:** 10.1055/a-2873-6868

**Published:** 2026-06-02

**Authors:** Michal M. Shalamov, Linsey R. Cromwell, Pallabi Guha

**Affiliations:** 1 Department of Pediatrics/Neonatology 23498 HMH K. Hovnanian Children’s Hospital at Jersey Shore University Medical CenterNeptune CityNew JerseyUnited States

**Keywords:** congenital central hypoventilation syndrome, *PHOX2B*, apnea, hypercapnia, hypoxemia, autonomic dysfunction

## Abstract

**Background:** Congenital central hypoventilation syndrome (CCHS) is a rare genetic disorder that causes alveolar hypoventilation because of impaired chemoreceptor response to hypercapnia and hypoxia occurring due to a dysfunctional central autonomic drive. CCHS is a result of pathogenic variants of the *PHOX2B* gene, which affects neural crest cell development. Consequently, CCHS can be accompanied by dysregulation of the autonomic system, Hirschsprung’s disease and other gastrointestinal disorders, cardiac arrhythmias, and neural cell-derived tumors. Disease severity correlates with mutation type and determines ventilatory requirements, with management centered on lifelong respiratory support and multidisciplinary monitoring for associated comorbidities.

**Case Presentation:** This case describes a term infant delivered via emergency cesarean section for maternal hypotension and fetal bradycardia who presented with apnea and required intubation due to failure to respond to positive pressure ventilation. The infant experienced recurrent apnea, hypercarbia, and ventilator dependence due to multiple failed extubation attempts, with an unrevealing evaluation for pulmonary, cardiac, neurologic, metabolic, and infectious causes. Genetic testing identified a *PHOX2B* polyalanine repeat expansion consistent with CCHS. This case demonstrates a stepwise multidisciplinary approach with concurrent genetic evaluation that enabled early diagnosis, timely management, and improved outcome.

## Case

### Maternal History

A female infant was born to a 26-year-old G2P1001 mother at gestational age of 38 weeks and 4 days. The mother’s prenatal labs were unremarkable, including a group B *Streptococcus* -negative status. Maternal prenatal history was significant for iron deficiency anemia, sleep apnea, and polycystic ovarian syndrome. Her surgical history included a cholecystectomy and a gastric bypass.

### Delivery

The infant’s mother presented in labor, which was subsequently complicated by hypotension and fetal bradycardia. The mother was taken for an emergency cesarean section under general anesthesia due to concern for amniotic fluid embolism.

### Presentation at Birth

The infant was born with some tone and respiratory effort at delivery; however, she exhibited persistent intermittent apnea and hypoxia requiring positive pressure ventilation and subsequent intubation. APGAR scores were 7 and 8 at 1 and 5 min of life, respectively.

### Neonatal Intensive Care Unit Clinical Course

The infant was transferred to the neonatal intensive care unit (NICU) for respiratory failure. The patient’s non-reassuring fetal heart rate tracing and intubation were initially concerning for hypoxic–ischemic encephalopathy (HIE). However, the infant had respiratory effort at birth followed by secondary apnea, which is not consistent with HIE/perinatal event. Moreover, the infant had a normal neurologic exam and cord gases, and thus did not meet the criteria for therapeutic hypothermia.

The infant was extubated to CPAP (Continuous Positive Airway Pressure) +5 at FiO _2_ (Fraction of Inspired Oxygen) 40% on day of life (DOL) 0. She received an echocardiogram on DOL 2, revealing severe persistent pulmonary hypertension of the newborn and a patent ductus arteriosus. FiO _2_ was then titrated to 100%. She improved clinically over the next 24 hours, followed by decompensation with increased work of breathing and desaturations, therefore requiring reintubation on DOL 3. By DOL 4, the infant exhibited no increased work of breathing, stable blood gas analyses, and was clinically improving. She was extubated again to CPAP. However, by evening, she had a sudden onset of lethargy and significant pallor, prompting an evaluation that included a blood gas analysis that revealed hypercapnia to 103 mmHg. She was reintubated with significant improvement and normocapnia. Over the next 24 hours, she had periods of normal blood gas analyses prompting weaning of the ventilator, followed by recurring hypercapnia leading to a concern for persistent apnea.

Pulmonology was consulted to investigate for hypoventilation syndromes, and caffeine was started for possible apnea of prematurity, although she was born full-term.

Neurology was consulted: Magnetic resonance imaging (MRI), angiography, and venography were obtained and found to be normal. Video electroencephalography (vEEG) was started and demonstrated occasional sharp waves in the right central head regions, which at times formed brief runs without evolution into electrographic seizures. The infant was, therefore, started on phenobarbital, given the clinical suspicion for seizures in the setting of apneic episodes and vEEG abnormalities. The antiepileptic was discontinued on DOL 21 with improvement of electrographic activity on vEEG.

Genetics was consulted, and testing included chromosomal micro-single nucleotide polymorphism array, urine organic acids, plasma acylcarnitines, homocysteine, urine acylglycines, urine purine/pyrimidine panel (all within normal limits), respiratory distress panel (showed heterozygosity of *MSK1* gene, which was analyzed by genetics and deemed to be nondiagnostic), congenital central hypoventilation panel (was *positive* ), and further PGxome RAPID Exome and respiratory distress panels were positive for *PHOX2B* gene.

The infant continued to have four total planned and unplanned extubations, NIMV (Non-Invasive Mechanical Ventilation) trials, and subsequent reintubations. A tracheostomy and gastrostomy tube were placed on DOL 49 with a 3.5-mm NeoShiley placement. The infant further required aggressive pulmonary toileting with chest physical therapy, pulmozyme, albuterol every 8 h, frequent suctioning, and repositioning. Persistent pulmonary hypertension eventually resolved without requiring nitric oxide or sildenafil. The infant was discharged to a children’s specialized rehabilitation hospital with continued multidisciplinary follow-up.

Otolaryngology, gastroenterology, surgery, cardiology, sleep medicine, speech, physical, and occupational therapy eventually became involved in the infant’s management. At 15 months of age, she was able to be weaned to room air during the day but required continued mechanical ventilation in a volume control mode during sleep via her tracheostomy. Her gastrostomy tube was taken out at 14 months of age, and the patient is now able to eat a normal diet.

### Differential Diagnosis

Neuromuscular disorder

Brainstem lesions

Pulmonary disorder

Cardiac disorder

Aspiration

Infection/sepsis

Metabolic disease

Myelomeningocele

### Actual Diagnosis

Congenital central hypoventilation syndrome

## Discussion

Congenital central hypoventilation syndrome (CCHS) is a rare autonomic nervous system (ANS) disorder affecting the function and control of breathing. 1 It is characterized by hypoventilation and shallow breathing during sleep (especially in nonrapid eye movement sleep) and sometimes also when awake. It is caused by mutations of the *PHOX2B* gene, which also affects the development of neural crest-derived structures. Therefore, CCHS can affect the ANS and be associated with Hirschsprung’s disease and the development of tumors that are neural crest in origin. Moreover, neurocognitive delay can also occur from hypoxic events that arise from a diminished respiratory function. 1 2

CCHS is a diagnosis of exclusion and should be suspected in newborns with no evidence of a primary lung, cardiac, brain, or other neuromuscular disease (negative MRI brain and brainstem, chest radiography and echocardiography, airway endoscopy, metabolic testing, and neurological evaluation), who are demonstrating unexplained shallow breathing, hypoventilation, and an abnormal ventilatory response to hypercarbia or hypoxemia when asleep and sometimes also when awake. 2 3 Neonates will not show the typical clinical signs of hypercarbia and hypoxemia, such as tachypnea or chest retractions, and therefore need to be continuously monitored with pulse oximetry and capnography. The main physiologic cause of CCHS is alveolar hypoventilation, which results in the inability to mount an appropriate respiratory response to hypercapnia and hypoxemia. Neonates show hypoventilation, apnea, hypercarbia, hypoxemia, difficulty with extubation, and transitioning off artificial ventilation, which can, in turn, result in pulmonary hypertension. 1 A similar pattern was noted in our case, as our patient exhibited apneas due to a depressed ventilatory drive leading to hypercapnia upon several extubation attempts. Echocardiography showed pulmonary hypertension upon admission, which eventually resolved. Today, our patient continues to follow up with cardiology yearly.

CCHS can cause ANS dysregulation, leading to decreased heart rate, sinus pauses, cyanotic breath-holding spells, lack of normal physiologic response to exercise and environmental stressors, as well as gastrointestinal disorders, such as esophageal dysmotility, constipation, profuse sweating, lack of perception of anxiety, dysregulated body temperature, and reduced pupillary light response. 4 Although our patient initially required gastrostomy tube support during her NICU course and after discharge, the tube was successfully removed at 14 months of age. She now tolerates a regular oral diet and has normal daily bowel movements.

CCHS causes a lack of appropriate physiologic arousal from sleep during physiologic hypercarbia and hypoxemia. At 14 months, our patient underwent a ventilatory titration sleep study, which demonstrated adequate respiratory support with normal carbon dioxide levels. She remains on room air during the day and has transitioned to volume-control ventilation required only during sleep and naps, when intermittent apneic episodes do occur.

As CCHS-affected neonates develop, they may experience neurocognitive decline due to cerebral compromise, repetitive states of hypoxia arising from the need for constant artificial ventilation, breath-holding causing cyanosis, sinus pauses, seizures, hypercarbia due to poor ventilation, ANS dysfunction affecting brain function, and structural brain abnormalities related to the *PHOX2B* gene mutation. 2 A 2016 retrospective review by Charnay et al. examined preschoolers with confirmed CCHS, aged 42 weeks and under, as they underwent developmental testing with a version of the Bayley Scales of Infant Development between the years 1993 and 2013. They showed that, on average, compared to population norms, preschoolers diagnosed with CCHS showed decreased developmental abilities in both motor and developmental indices. However, they also indicated marked variability in the indices between the CCHS preschoolers, ranging from 48 to 138. 5

In this case, early involvement of pulmonology and genetics during our patient’s NICU course facilitated timely diagnosis and contributed to a favorable neurodevelopmental outcome. At present, the patient demonstrates age-appropriate development with normal Survey of Well-being of Young Children (SWYC) scores. She continues to receive supportive therapies, including monthly speech therapy, weekly physical and occupational therapy, and developmental intervention three times per week.

In order to establish a diagnosis of CCHS, genetic and molecular testing must identify and confirm a heterozygous *PHOX2B* pathogenic variant, and patients must demonstrate clinical findings suggestive of hypoventilation. Genetic evaluation is also important in order to clarify the risk of other family members and offspring from having CCHS. CCHS is inherited in an autosomal dominant pattern of the *PHOX2B* gene and usually caused by a de novo pathogenic mutation, although somatic or germline mutations have been found in affected individuals with asymptomatic parents. A high risk of parental mosaicism exists; therefore, once an affected child has been identified, preimplantation genetic testing and/or prenatal testing is recommended for future pregnancies. However, most cases involve a single affected family member. 2 There are three types of *PHOX2B* pathogenic variants: Polyalanine repeat expansion mutations (PARMs), non-polyalanine repeat expansion mutations (NPARMs), and exon 3 or whole-gene deletions (a subset of the NPARMs). 2 In general, NPARM mutations cause more severe disease and phenotypes, which result in the need for constant artificial ventilation, as well as a higher risk of neural crest tumors such as neuroblastoma, ganglioneuroblastoma, ganglioneuroma, and Hirschsprung’s disease. Notably, Hirschsprung’s disease and other gastrointestinal disorders such as gastroesophageal reflux, dysmotility, and dysphagia can be a manifestation of PARM mutations as well. 1 6 7

In this case, the patient was found to have a de novo PARM mutation of *PHOX2B* . PARM mutations are associated with decreased transcription of genes. Patients may present at any age from neonatal to adulthood. The length of the PARMs expansion of the alanine amino acids in the *PHOX2B* gene determines the severity of the disease. 6 In our case, CCHS was a congenital diagnosis, and the patient was found to have 25 repeats of PARM, and familial genetic testing was negative.

The management of CCHS includes a multidisciplinary approach, including sleep medicine, neurology, pulmonology, cardiology, gastroenterology, oncology, ophthalmology, neurodevelopmental psychology, otolaryngology, and genetics. Treatment often involves artificial ventilation and airway protection such as tracheostomy, positive pressure ventilation, diaphragmatic pacing (placement of phrenic nerve electrodes), cardiac pacemakers, and protective eye wear. 1 2 6 Yearly chemistry count, blood gas, cell count, echocardiography, electrocardiography, neurocognitive assessments, autonomic dysfunction assessments, and sleep studies are recommended. 2 Moreover, patients should also avoid swimming due to the risk of breath-holding, asphyxia, and death, as well as avoid the use of recreational drugs, ingestion of alcohol, or the use of prescription medication, such as sedatives and anesthetics, due to respiratory depression. 2

## Conclusion

This case demonstrates the clinical complexity and diagnostic course taken to identify CCHS in this infant. Our patient had numerous red herrings; we considered explanations for her symptoms along her clinical course, such as HIE, other neonatal causes of encephalopathy, apnea of prematurity, cardiac causes, brain and spinal cord lesions, sepsis, metabolic diseases, and other neuromuscular etiologies, which had to be ruled out. We, therefore, highlight the importance of the involvement of a multidisciplinary team promptly to ensure a quicker diagnosis and thus appropriate management of affected patients. Concurrent investigation of genetic causes and early diagnosis of CCHS prevents fatal respiratory failure, minimizes cerebral injury secondary to hypoxia, and ensures immediate initiation of life-saving support. This ultimately resulted in a prompt diagnosis of CCHS and thus ensured our patient’s survival and improved quality of life.
